# Ontogeny of rake mark scarring on common dolphins (*Delphinus delphis*): insights to aggression and affiliation?

**DOI:** 10.1098/rsos.252175

**Published:** 2026-10-14

**Authors:** Hannah R. Crisp, Katharina J. Peters, Emily I. Palmer, Frédérik Saltré, Emma L. Betty, Eva-Maria F. Hanninger, Karen A. Stockin

**Affiliations:** ^1^ Cetacean Pathology Unit, New Zealand Institute for Advanced Study, Massey University, Auckland, New Zealand; ^2^ Marine Vertebrate Ecology Lab, Environmental Futures, School of Science, University of Wollongong, Wollongong, New South Wales, Australia; ^3^ Biogeography Ecology and Modelling | Ngura Nandamari, School of Life Sciences, University of Technology Sydney, Sydney, New South Wales 2007, Australia; ^4^ Australian Museum Research Institute, Australian Museum, Sydney, New South Wales 2010, Australia

**Keywords:** aggression, affiliation, life history, scarring, social biology, post-mortem

## Abstract

Aggressive and affiliative interactions shape resource allocation, social structure and reproduction in social species. In delphinids, rake marks (parallel scars from tooth raking) indicate social interactions. When examined alongside ontogeny, demographic traits and scar position, rake marks can inform social dynamics. We assessed rake marks on common dolphins (*Delphinus delphis*) in Aotearoa New Zealand (2006–2024; *n* = 102) during post-mortem examinations to explore drivers of social behaviour. Using a zero-inflated generalized linear mixed model, we assessed rake mark occurrence (probability of rake density > 0) and prevalence (rake density, rake marks dm^−2^) across the body (*n* = 101; dorsal/ventral, cranial/caudal, left/right) in relation to sex, age, total body length (TBL), sexual maturity, body condition (*n* = 101) and female reproductive status (*n* = 51), to infer confrontation, avoidance and lateralization. Females showed higher rake mark prevalence but lower occurrence than males, suggesting infrequent but prolonged interactions, possibly sexual coercion. Rake prevalence and occurrence increased with TBL, potentially related to increased social interactions and surface area in larger individuals. Age, sexual maturity, body condition, and female reproductive status were not strong predictors of rake mark occurrence or prevalence. Our findings highlight rake marks as potential indicators of social dynamics.

## 1. Introduction

Social living in mammals provides clear benefits, including cooperative resource sharing [1], predator protection [2] and opportunities for affiliative interactions such as play and social touch, that may lead to enrichment and social bonds [3,4]. However, group living also carries costs such as conflict and aggression, which can disrupt beneficial relationships [5] and present energetic, physical and physiological burdens [6,7]. This presents potential consequences that may impact health and individual well-being [8–10].

Delphinids are highly social mammals, interacting in a wide variety of contexts including cooperation [11], affiliative play [12], socio-sexual behaviour [13] and competition [11]. Such interactions shape their social structure [14–16], resource allocation [7,14,17,18] and reproductive success [12,16,19–21].

Similar to other delphinids, common dolphins (*Delphinus delphis*) exhibit social complexity, with populations around the world displaying diverse social structures and behaviours, including fission–fusion dynamics [22–25] and strong associations [26–28]. In Aotearoa New Zealand, common dolphins display high variability in group size [11,24,29–31], and form lasting social bonds, as evidenced by long-term preferred associations among individuals [24,26]. Despite their social complexity, studies examining common dolphin behaviour remain limited and primarily restricted to captive studies or field observations. In part, this is because direct observation of delphinid social behaviour is challenging owing to the discrete nature of their interactions and their predominantly submerged aquatic lifestyle [32]. However, past interactions can be inferred via body scarring. This approach has been used in studies of rake scarring on a range of delphinid species [14,17,19,21,33,34]. Rake marks, semi-permanent wounds or scars which appear as shallow, parallel indentations or lines caused by delphinid teeth dragging across the dermis and/or sub-dermis, provide a quantifiable measure of social interactions in delphinids [14,16,17,21,35,36].

Rake marks are known to occur in the context of aggressive interactions between delphinids, such as intra- and inter-specific competition, sexual coercion and predation [16,17,37–40]. For example, aggressive posture, sound and movements, including chasing, body slamming, ramming and biting [16,41] are typical, often in the context of competition for resources [11,42,43], mating opportunities [16,17,44,45], defense of self and offspring [34,46–48] and the establishment or maintenance of dominance hierarchies [28,49]. Aggression within social groups can have adverse effects, potentially affecting delphinid social structure [14–16], reproductive success [19–21] and resource allocation [7,14,17,18].

Alternatively, rake marks in some cases have also resulted from non-aggressive social interactions among delphinids [16–18]. In particular, raking has been associated with affiliative socio-sexual and play behaviour, which involves chasing, mouthing (rubbing jaw and opening and closing mouth near one another), biting and rough-and-tumble behaviours such as rolling and wrestling [35,50]. Such interactions are important for the development of social bonds, and the transmission of social behaviours [35]. Accordingly, rake marks should not be viewed exclusively as indicators of aggression, but rather as evidence of a broad range of interactions and behaviours that occur in a range of social contexts.

Rake marks heal over time, as observed in bottlenose dolphins, where healing typically occurs within 400 days [7,17]. Therefore, such marks may signal a range of different social behaviours for an individual over the time period in which healing occurs. Nonetheless, the assessment of rake marks allows insight into the occurrence and inferred context of social interactions that might otherwise be missed [14,16,45].

Measuring inter-rake distances (i.e. the distance between parallel indentations or lines within the rake marks), can identify species based on dentition spacing [33,37,39,51]. In addition, pairing rake mark data with traits such as age, total body length (TBL), sex, sexual maturity, reproductive status and body condition can contextualize social interactions [17,51]. Furthermore, the distribution of rake marks across the body may also assist in discerning the origin and nature of the interactions, such as distinguishing between competition, sexual behaviour or play [35]. Rake mark positioning can further be used to investigate behavioural lateralization in delphinids, to determine whether certain sides of the body are more frequently targeted during encounters, potentially correlating with observed behavioural patterns [52]. However, it remains unclear whether such lateralization reflects the preference of the initiators, or a response by the recipient (i.e. bias as to which side of the body is presented to an attacker during interactions).

To date, the assessment of rake marks on common dolphins in New Zealand in the published literature is limited to field-based studies that use opportunistic photographic identification data to assess rake mark prevalence [53,54]. Such studies are often restricted to recording rake marks on the dorsum, particularly the dorsal fin. In contrast, observation of rake marks on fresh cadavers allows analysis of rake mark occurrence, frequency and position to be assessed across the entire body surface, in conjunction with associated life-history data. With the correct quality assurance in place, this can provide quantifiable evidence of recent social interactions and offer new insights into reproductive strategies, social structures and health of delphinids [16,17,34,36,39].

Here, we examine the occurrence (presence/absence) and prevalence (rake density) of conspecific rake marks on New Zealand common dolphin cadavers in the context of ontogeny and scarring position to investigate potential drivers of social interactions. Specifically, we assess whether rake density varies based on sex, age, body length, sexual maturity, female reproductive status, body condition and body sector. We further analysed the distribution of rake marks across the body and their correlation with these biological parameters. This provides the first assessment of how ontogeny and demographic traits influence rake mark scarring in common dolphins in New Zealand waters.

## 2. Material and methods

We examined records from 102 common dolphins examined during necropsies between 2006 and 2024, following standardized post-mortem methods [55,56]. Dolphins were included in the study if high-quality photographs of each body surface were available, and/or if lesions (herein referring to markings on the skin including but not limited to rake marks) and skin loss were recorded. Inclusion also required the following metadata: age, sex, sexual maturity, reproductive status, body morphometrics, body condition and decomposition stage. Individuals for which age was unknown, but all other variables were known, were also retained. Temporal factors such as season and year were not included in the analyses, as stranding time and location may not accurately represent the timing or location of the behavioural interactions that resulted in rake marks.

### 2.1. External examination

Each cadaver was examined grossly for rake marks across the entire body surface. Photographs were taken of detected lesions using a scale bar and a grey scale marker. For each rake mark, we recorded the body sector (dorsal head, ventral head, dorsal cranial flank, ventral cranial flank, dorsal fin, dorsal caudal flank, ventral caudal flank, dorsal peduncle, ventral peduncle, pectoral fins and fluke—figure 1), lesion length (cm), lesion width (cm) and inter-rake distance (mm). Post-mortem skin loss from transportation and decomposition was visually estimated for each body sector as a percentage. Body morphometrics, including body length, were additionally recorded (supplementary material, table S1). Body condition, used as a proxy of health, was assessed as good, moderate or poor based on standardized morphological criteria (supplementary material, table S2). Decomposition stage was assessed as fresh, mild, moderate or severe, following Stockin *et al.* [56] (supplementary material, table S3). All cadavers included in the study retained sufficient skin integrity to reliably detect rake marks. Specifically, individuals were excluded if skin sloughing or degradation (decomposition stage moderate and severe) of the body surface could potentially obscure external markings.

**Figure 1.**
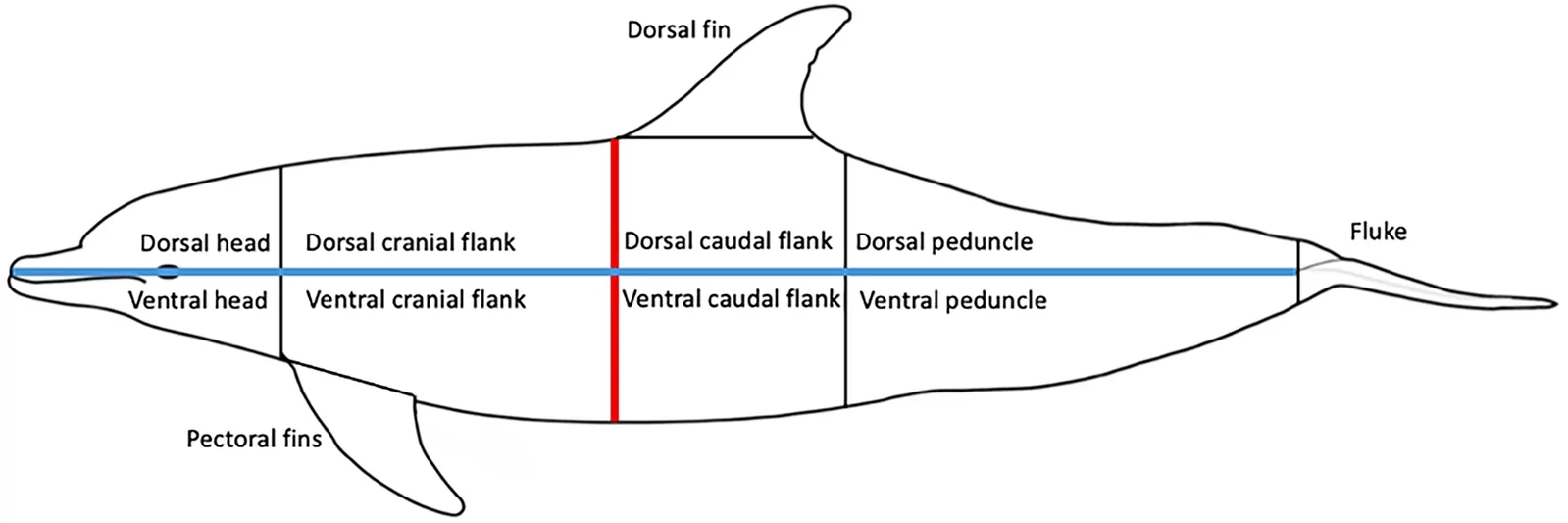
Body sectors used to record rake mark position on common dolphin (*Delphinus delphis*) cadavers. Pooled sector groupings show dorsal grouping above the blue line, ventral below, cranial to the left of the red line and caudal to the right (modified from [16,21,53]).

### 2.2. Rake mark location and characteristics

We evaluated rake marks across 11 body sectors (figure 1), and recorded their presence for orientation (dorsal, ventral, lateral left/right). Lesions occurring across two sectors were assigned to the sector where the majority (more than 50%) of the lesion occurred. Only lesions with multiple, uniform, parallel indentations or lines were classified as rake marks. Rake marks were included in analyses if the inter-rake distance was approximately 5 mm, consistent with conspecific interactions. Rake marks with inter-rake distances greater than 10 mm were excluded owing to probable interspecific origin. For analysis, sectors were pooled into dorsal, ventral, cranial, caudal, left and right body sector groupings (figure 1).

### 2.3. Age estimation

Teeth were extracted, decalcified, sectioned and stained for age estimation following Palmer *et al.* [57]. Annual growth layer groups (GLGs) in dentine, each representing one year, were counted to estimate minimum age [58,59]. Two readers independently counted GLGs blind (i.e. with no prior biological knowledge) to verify the estimates [57].

### 2.4. Sex, sexual maturity and reproductive status

Sex and sexual maturity were determined by gross and histological gonad examination [57,60]. Males were classified as sexually immature if seminiferous tubules contained abundant interstitial tissue and minimal spermatogonia. Pubescent males displayed spermatogonia and spermatocytes, larger seminiferous tubules and reduced interstitial tissue. Sexual maturity was characterized by the presence of active spermatogenesis (spermatids and spermatozoa) [60].

Females were considered sexually mature if pregnant (foetus in the uterus and a *corpus luteum* on an ovary), lactating (milk in mammary glands) or *corpora albicantia* and/or *corpora lutea* were present on the ovaries [57]. Female reproductive status was assessed through examination of the ovaries, uteri and mammary glands, and categorized as: (1) sexually immature, *corpora albicantia* and/or *corpora lutea* absent from ovaries; (2) pregnant and lactating, when a foetus is present, a *corpus luteum* is observed on one ovary and milk is present in the mammary glands; (3) lactating, milk production is evident in the mammary glands; and (4) resting mature, for sexually mature females not currently pregnant or lactating [57].

### 2.5. Data analysis

Rake density, defined as the number of rake marks (rm) per dm² (10 cm × 10 cm) of skin available, was calculated to standardize the measurement of rake marks across dolphins with different surface areas and varying degrees of skin loss and/or decomposition. The area of each sector was calculated from morphometric measurements (supplementary material, fig. S1). The cranial and caudal flanks were measured using the area of a rectangle formula (length × width). The head and peduncle sectors used the area of a triangle formula (0.5 × base × height).

Adobe Photoshop pixel tracing was used to determine the areas of the dorsal fin, pectoral fins and fluke owing to their irregular shapes. Each sector was manually outlined using the tracing tool, and the software calculated sector areas by integrating pixel counts with corresponding body morphometrics to account for scale. A resampling technique estimated areas for irregularly shaped sectors where individuals did not have images suitable for pixel tracing. Leave-one-out cross-validation was used to validate area estimates. The ‘area of skin visible’ was calculated by subtracting the percentage of skin loss from the corresponding area. The rake density (rm dm^–2^) was then calculated for each sector, sector grouping and the entire body using the following formula:

$$\begin{array}{r} Rake\;density=\frac{Rake\;count}{Area\;of\;skin\;visible}. \end{array}$$

Descriptive statistics (mean, standard deviation, minimum and maximum) were calculated for rake density across sex, sexual maturity, female reproductive status and body condition. Owing to limited sample sizes, the reproductive status categories ‘pregnant’, ‘lactating’ and ‘pregnant and lactating’ were pooled into a single category, ‘pregnant and/or lactating’. Age and body length were plotted with rake density using scatterplots with a regression line. Descriptive statistics of rake density were also calculated for each body sector and sector grouping. One individual was missing age data owing to its teeth being unavailable and was therefore excluded from the multivariate analysis, but included in the descriptive statistics for variables except age. Zero-inflated models are useful for analysing semi-continuous, heteroscedastic data with a high proportion of zeros and a positive skew of non-zero values, which commonly occur in biomedical and ecological research [61–63], including the rake density data used in this study. Zero-inflated generalized linear models (ZIGLMs) [64] were used in R version 4.3.3 [65] to assess the effect of age, body length, sex, sexual maturity, female reproductive status, and body condition on rake density.

Zero-inflated generalized linear models included two components: a gamma conditional model and a zero-inflation model. The gamma component modelled variation in positive, non-zero rake density values, estimating how rake density changed when rake marks were present. The zero-inflation component modelled the occurrence of zeros, estimating the probability that rake marks were absent.

We also used zero-inflated generalized linear mixed-effects models (ZIGLMMs; [66] to compare rake density across body sectors while testing the effects of age, body length, sex, sexual maturity and body condition. We included individual ID as a random effect to account for repeated measurements across sectors.

Body sector effects were analysed in three independent models (dorsal versus ventral, left versus right, and cranial versus caudal) because each plane was compared separately to test different biological hypotheses regarding the distribution of rake marks. To assess the independent contribution of each predictor variable, models including each sole predictor were evaluated alongside models with a combination of predictors.

Biologically relevant interaction terms were considered to account for dependencies among predictor variables, as the occurrence and accumulation of rake marks are probably influenced by life-history stage, morphology, sex and body condition. The relationship between age and rake density may differ with sex owing to sex-specific social behaviours and the onset of sexual maturity. TBL was also expected to interact with sex and sexual maturity, reflecting sexual size dimorphism and its potential influence on dominance and exposure to aggressive and socio-sexual interactions. Body condition was considered in interaction with age, reproductive status and sexual maturity, as energetic demands associated with growth, reproduction and ageing may influence both social interactions resulting in rake marks, as well as healing capacity. Female reproductive status was further examined in interaction with age, TBL and sexual maturity, as pregnant and/or lactating individuals may experience different social pressures and energetic constraints compared to non-reproductive individuals. Interaction terms were included where biologically justified and if they improved model fit based on Akaike information criterion corrected for small sample sizes (AICc) and AICc weights [67].

Where needed, response variables were square-root transformed to meet model assumptions. Results were presented on the original scale, with exponentiated coefficients (exp(Est)) calculated for interpretation. We assessed model diagnostics using residual plots and checked for heteroscedasticity, multicollinearity, residual uniformity and, where applicable, normality of random effects. When we detected heteroscedasticity, we tested square-root transformations of rake density to improve variance stabilization. Because transformed and untransformed models led to the same biological conclusions, we report results from the untransformed models on the original rake density scale. Where appropriate, we calculated exponentiated coefficients, exp(Est), to aid interpretation. We visualized model outputs with effect size plots showing how predictor variables affected rake mark density and occurrence.

## 3. Results

### 3.1. Sample summary

A total of 102 common dolphins examined between 2006 and 2024 were included in the descriptive analysis of rake density (rm dm^−2^), including 56 and 46 females and males, respectively. Rake marks were observed on 79.4% of examined cadavers (*n* = 81), including 45 females and 36 males. Of the females with rake marks, 17 were sexually immature and 28 were sexually mature. Of the males, 19 were sexually immature and 17 were sexually mature. A further 21 individuals showed no rake marks. Rake density across the full sample (*n* = 102) ranged from 0 to 1.06 (mean ± s.d. = 0.17 ± 0.20). To assist with the interpretation of rake density values, we provide a representative figure of scanned post-mortem rake mark data sheets, recorded during post-mortem assessments, illustrating the range of rake densities observed in our dataset (figure 2). Supplementary scanned data sheets detailing lesion characteristics, including rake length (cm), width (cm) and inter-rake distance (mm), are provided in the supplementary material (fig. S2). One individual was missing age data. This resulted in 101 individuals (*n* = 80 with rake marks; *n* = 21 without rake marks) being included in the multivariate analysis.

**Figure 2.**
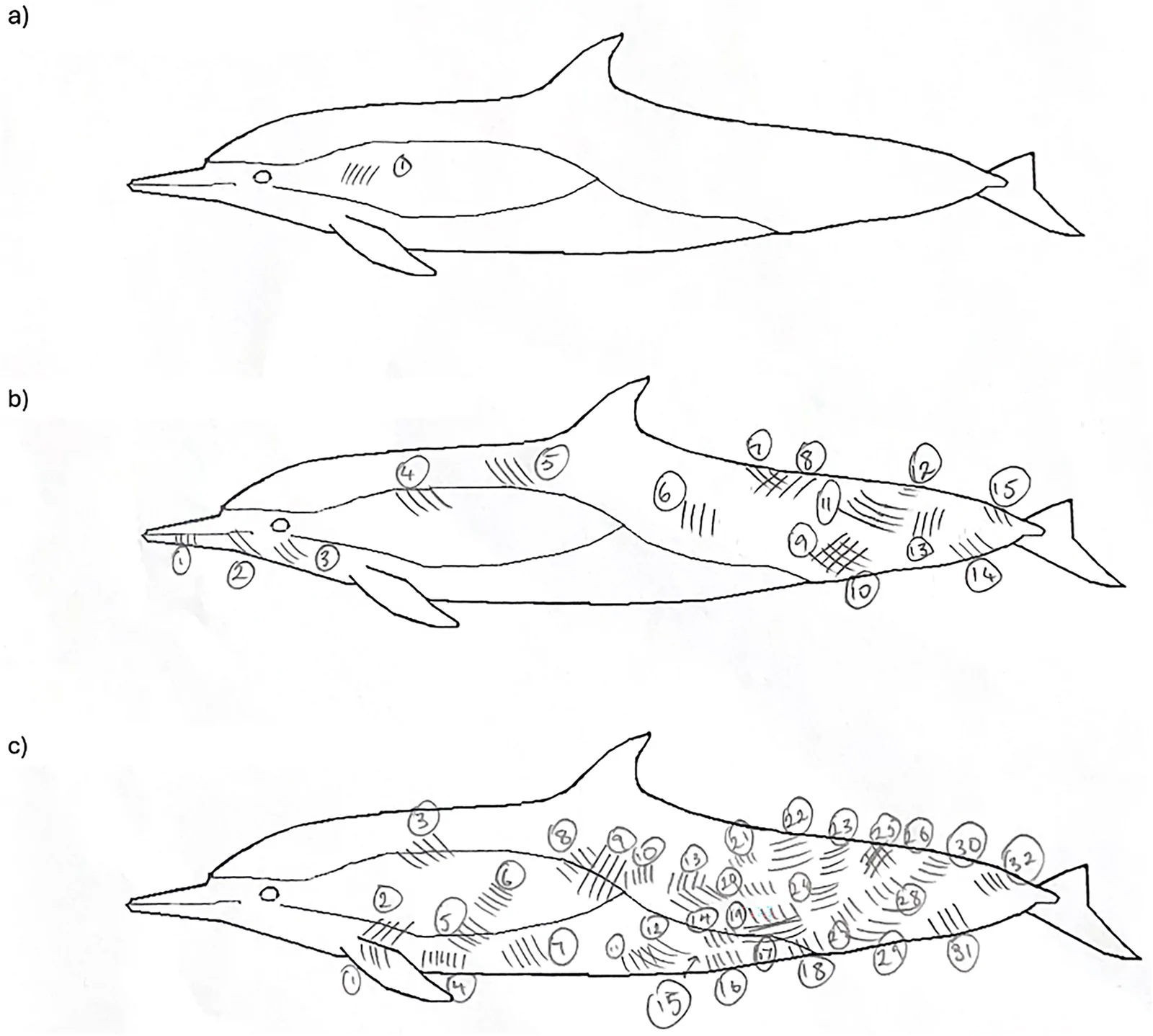
Exemplar of rake density recorded from post-mortem datasheets of common dolphin (*Delphinus delphis*) cadavers showing (a) low rake density (0.02 rm dm^−2^), (b) medium rake density (0.46 rm dm^−2^) and (c) high rake density (1.06 rm dm^−2^).

### 3.2. Rake mark occurrence and prevalence

#### 3.2.1. Descriptive statistics

Rake density ranged from 0 to 1.06 (mean ± s.d. = 0.20 ± 0.23, *n* = 56) and 0 to 0.60 (mean ± s.d. = 0.13 ± 0.15, *n* = 46) for females and males, respectively (supplementary material, table S4). Rake density also varied across sexual maturity, body condition and female reproductive status (supplementary material, table S4). Overall, females exhibited higher rake densities than males, with mature individuals showing higher densities than immature individuals. Within females, resting mature and pregnant and/or lactating individuals tended to have the highest rake densities, while immature females displayed the lowest values. In males, mature individuals generally had higher rake densities than immatures, although the difference was less pronounced than in females. Individuals with moderate body condition showed slightly higher rake densities than those in good condition (supplementary material, table S4).

Across all individuals with age data (*n* = 101), age ranged from 0 to 31 years (mean ± s.d. = 7.88 ± 7.23). For females and males, respectively, age ranged from 0 to 25 years (mean ± s.d. = 8.49 ± 6.66, *n* = 55) and 0 to 31 years (mean ± s.d. = 7.17 ± 7.87, *n* = 46). Visual inspection of the data indicated that rake density increased with age, with older individuals on average exhibiting a higher rake density than younger individuals (supplementary material, fig. S3). A similar pattern was observed with TBL, as larger individuals tend to show higher rake densities than smaller individuals (supplementary material, fig. S4).

#### 3.2.2. Multivariate analysis

Sex was the most important predictor of rake density, with females displaying higher rake densities than males (table 1). The conditional model indicated that males displayed approximately 40.6% lower rake density on average (*p* = 0.003; table 1). Rake density also increased with TBL, indicating that larger individuals tend to show higher rake densities than smaller individuals (*p*= 3.3 × 10^−13^; table 1). Females were shown to have a steeper increase in rake density with TBL compared to males (figure 3a).

**Table 1.** Best-fitting zero-inflated generalized linear model (ZIGLM) of rake mark occurrence and rake density (rm dm^−2^) across the entire body in common dolphins (*Delphinus delphis*) assessed post-mortem. The conditional component (A) models positive, non-zero rake density values, while the zero-inflation component (B) models the probability of zero rake density. This best-fitting model included sex (female or male) and TBL (in cm) as predictors. Also displayed are estimated coefficients for predictor variables (Est), their standard errors (SE), *p*-values, and the corresponding back-transformed estimates (exp(Est)).

model	variable	est	SE	*p*-value	exp(Est)
rake density ∼ Sex + TBL					
(A) conditional model:					
	(Intercept)	−6.64	0.72	<2.0 × 10^−16^	0.0013
	Sex_Male	−0.51	0.18	3.0 × 10^−3^	0.59
	TBL	0.03	3.8 × 10^−3^	3.3 × 10^−13^	1.03
(B) zero-inflation model:					
	(Intercept)	10.23	2.71	1.6 × 10^−4^	28 400.30
	Sex_Male	−0.83	0.91	0.36	0.44
	TBL	−0.08	0.02	4.3 × 10^−5^	0.92

**Figure 3.**
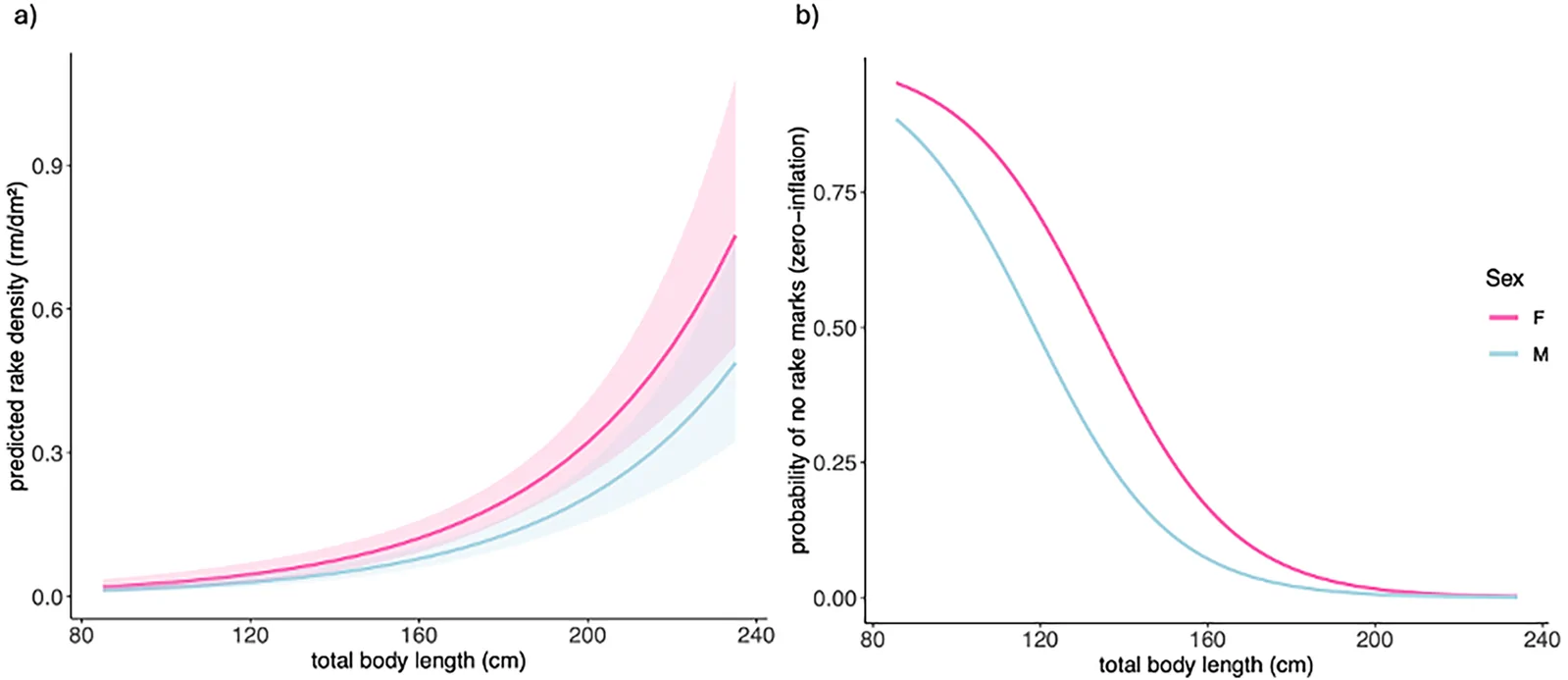
(a) Predicted rake mark prevalence (rake density (rm dm^−2^)) with TBL (cm) across the entire body in common dolphins (*Delphinus delphis*) assessed post-mortem, based on the best-fit zero-inflated generalized linear model (ZIGLM) of rake mark occurrence and prevalence, which includes sex and TBL as predictors. Lines represent the rake density predicted by the ZIGLM for females (pink) and males (blue), with shaded areas indicating 95% confidence intervals. (b) Predicted rake mark occurrence (the probability of no rake marks) with TBL from the same model, shown for females (pink) and males (blue).

The zero-inflated model, which assessed rake mark occurrence rather than rake density, showed that larger individuals were less likely to have no rake marks than smaller individuals. The odds of zero rake density decreased by approximately 7.7% with each cm increase in body length (*p* = 4.3 × 10^−5^; table 1). Although females were estimated to have 56.4% lower odds of rake marks than males, this effect was not statistically significant (*p* = 0.36; table 1). The probability of having no rake marks decreased with increasing TBL in both sexes, although males consistently exhibited a lower probability of zero rake marks than females (figure 3b).

Neither age, sexual maturity or body condition were retained in the top-ranked model, indicating no detectable relationship between these predictors and rake density or rake mark presence/absence (supplementary material, table S5).

The best-fit model analysing the effect of reproductive status on female rake density retained body length alongside reproductive status as predictor variables (supplementary material, table S6).

The conditional model showed that rake density was positively associated with body length, with each additional centimetre corresponding with a 3.1% increase in rake density (*p* = 8.81 × 10⁻^7^; table 2). Reproductive status was not a significant predictor in this component of the model, although predicted rake densities were slightly higher in resting females (*p* = 0.197; table 2) and lower in pregnant and/or lactating females relative to immature females (*p* = 0.989; table 2); these effects were not statistically significant. Effect size plots show broadly similar rake densities across immature, pregnant and/or lactating, and resting females (figure 4a). Rake density increased with TBL across all categories (figure 4a).

**Table 2.** Best-fitting zero-inflated generalized linear model (ZIGLM) of rake mark occurrence and rake density (rm dm^−2^) across the entire body in common dolphins (*Delphinus delphis*) assessed post-mortem. The conditional component (A) models positive, non-zero rake density values, while the zero-inflation component (B) models the probability of zero rake density. This model used a subset of the full dataset that only includes females, and the top-ranked model includes TBL (in cm) and female reproductive status (immature, pregnant and/or lactating and resting mature) as predictors. Also displayed are estimated coefficients for predictor variables (Est), their standard errors (SE), *p*-values, and the corresponding back-transformed estimates (exp(Est)).

model	variable	est	SE	*p*-value	exp(Est)
rake density ∼ reproductive status + TBL					
(A) conditional model:					
	(intercept)	−7.32	1.04	2.14 × 10^−12^	6.62 × 10^−4^
	Repro_Preg/lact	0.004	0.33	0.99	1.00
	Repro_Resting	0.52	0.41	0.20	1.69
	TBL	0.03	6.6 × 10^−3^	8.81 × 10^−7^	1.03
(B) zero-inflation model:					
	(intercept)	7.81	4.00	0.01	2470.00
	Repro_Preg/lact	0.69	1.70	0.69	1.98
	Repro_Resting	−18.67	2.43 × 10^4^	1.00	7.86 × 10⁻^9^
	TBL	−5.90 × 10^−2^	2.20 × 10^−2^	0.01	0.94

**Figure 4.**
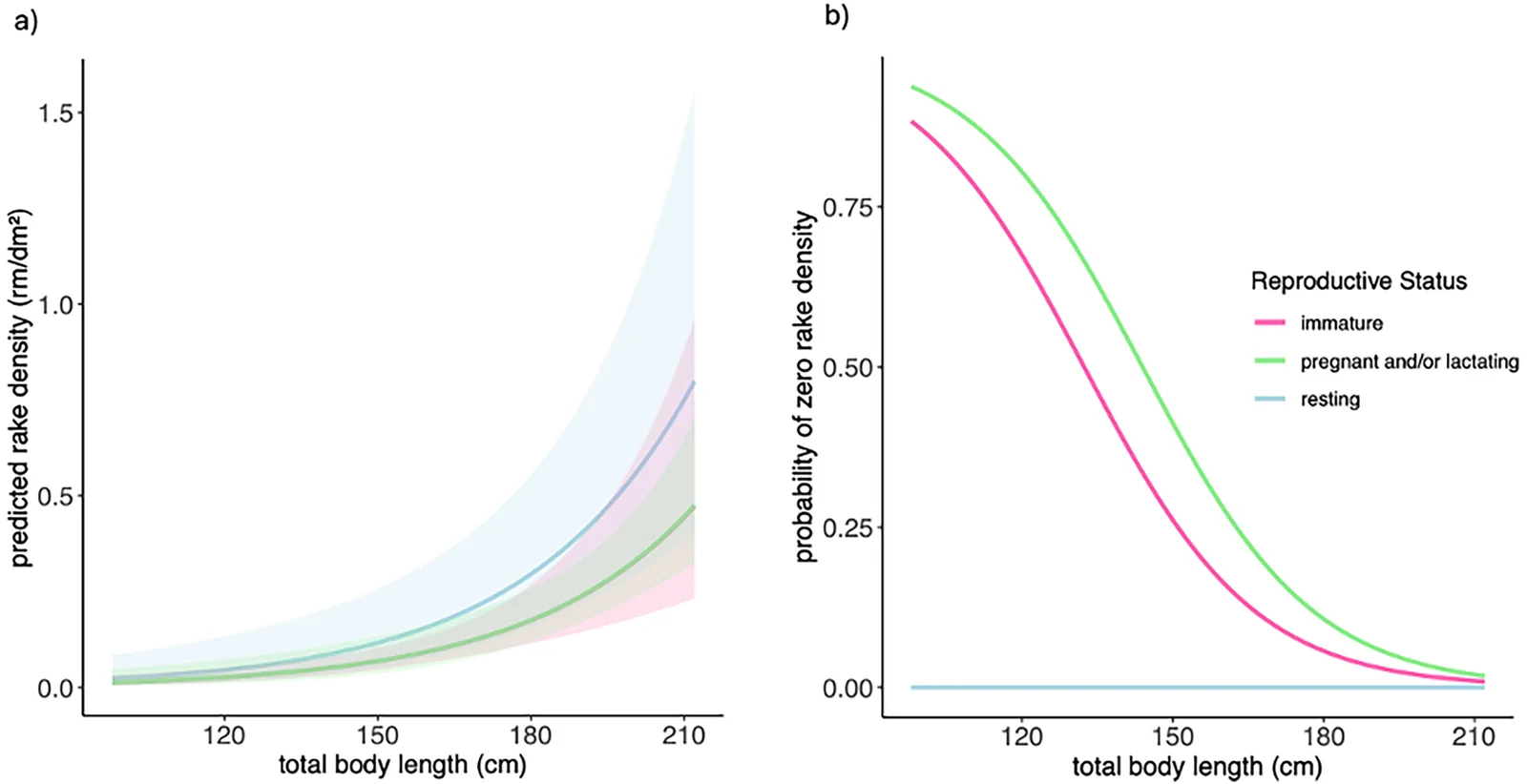
Predicted whole body rake density (rm dm^−2^) and the probability of zero rake density in relation to TBL (cm) in female common dolphins (*Delphinus delphis*) assessed post-mortem, based on the best fit zero-inflated generalized linear model (ZIGLM) including reproductive status and TBL as predictors. (a) Predicted positive rake density (rm dm^−2^) from the conditional component, shown for immature (pink), pregnant and/or lactating (green) and resting mature (blue) females. (b) Predicted probability of zero rake density from the zero-inflation component, shown for the same reproductive-status categories. Lines represent model predictions, and shaded areas indicate 95% confidence intervals.

The zero-inflation model showed that the probability of having no rake marks decreased with increasing body length (*p* = 0.007; table 2). It also indicated that, relative to immature females, pregnant and/or lactating females were less likely to have rake marks (*p* = 0.687; table 2). Estimates for resting mature females were unstable because no resting females had zero rake densities, limiting interpretation of this effect (*p* = 0.999; table 2). Both immature and pregnant and/or lactating females followed a similar declining trend in the probability of having no rake marks with increasing body length (figure 4b). However, these trends were not statistically significant, and zero inflation estimates for reproductive status were limited as resting females had no recorded zero rake densities (figure 4b).

Neither age, sexual maturity or body condition was retained in the top-ranked model, indicating no detectable relationship between these predictors and rake density or rake mark presence/absence (supplementary material, table S6).

### 3.3. Rake mark distribution and position

The right dorsal peduncle exhibited the highest mean and maximum rake density (mean ± s.d. = 0.60 ± 1.05) among body sectors, while the dorsal caudal flank displayed the lowest (mean ± s.d. = 0.00 ± 0.01). Among sector groupings, the caudal grouping showed the highest mean and maximum rake density (mean ± s.d. = 0.19 ± 0.22), with the cranial grouping having the lowest (mean ± s.d. = 0.13 ± 0.18).

#### 3.3.1. Lateral

For the left and right orientation, the best-fit model included body sector grouping, sex and body length as predictor variables, with an interaction effect between sector grouping and body length (supplementary material, table S7). The conditional model showed that males had a 29.5% lower rake density than females (*p*= 0.02; table 3a–i). Larger individuals demonstrated relatively higher rake density on the right-hand side, while smaller dolphins had higher rake density on the left (*p* = 0.0064 and *p*= 0.011 for sector and the interaction term, respectively; table 3a–i, figure 5a).

**Table 3.** Best-fitting zero-inflated generalized linear mixed models (ZIGLMMs) of rake mark occurrence and prevalence (rake density, rm dm^−2^) across body sector groupings in common dolphins (*Delphinus delphis*) assessed post-mortem. In each model, the conditional component (i) models positive, non-zero rake density values, while the zero-inflation component (ii) models the probability of zero rake density. These models include individual ID as a random effect. The lateral model (a) includes sex, TBL (in cm) and sector grouping (left versus right) as predictors, with an interaction between sector grouping and TBL. The dorsum-ventrum model (b) includes TBL, and sector grouping (dorsal versus ventral) as predictors, with an interaction between sex and sector grouping. The cranial versus caudal model (c) includes age, sex, TBL and sector grouping (cranial versus caudal) as predictors, with an interaction between TBL and sector grouping. Also displayed are estimated coefficients for predictor variables (Est), their standard errors (SE) and *p*-values, and the corresponding back-transformed estimates (Exp(Est)).

model	variable	est	SE	*p*-value	Exp(Est)
**(a) left** versus **right:** rake density ∼ sex + sector grouping * TBL + (1 | ID)					
**(i) conditional model:**					
	(intercept)	−4.03	0.58	4.96 × 10^−12^	0.02
	Sex_Male	−0.35	0.15	0.02	0.70
	Sector_Right	−1.83	0.67	6.36 × 10^−3^	0.16
	TBL	0.01	3.10 × 10^−3^	3.44 × 10^−5^	1.01
	Sector_Right:TBL	0.01	3.48 × 10^−3^	0.01	1.01
**(ii) zero-inflation model:**					
	(intercept)	7.81	2.43	1.30 × 10^−3^	2.46 × 10^3^
	Sex_Male	−0.09	0.57	0.88	0.92
	Sector_Right	6.13	3.00	0.04	459.34
	TBL	−0.05	0.02	8.46 × 10^−4^	0.95
	Sector_Right:TBL	−0.04	0.02	0.02	0.96
**(b) dorsal versus ventral:** rake density ∼ TBL * sector grouping + (1 | ID)					
**(i) conditional model:**					
	(intercept)	−4.93	0.54	<2.0 × 10^−16^	0.007
	Sector_Ventral	−1.07	0.73	0.15	0.34
	TBL	0.02	0.003	5.47 × 10^−9^	1.02
	Sector_Ventral:TBL	0.006	0.004	0.14	1.01
**(ii) zero-inflation model:**					
	(intercept)	7.59	2.80	8.64 × 10^−4^	1984.24
	Sector_Ventral	6.87	3.19	0.03	963.46
	TBL	−0.06	0.02	3.78 × 10^−4^	0.95
	Sector_Ventral:TBL	−0.04	0.02	0.06	0.96
**(c) cranial versus caudal:** rake density ∼ age + sex + TBL * sector grouping + (1 | ID)					
**(i) conditional model:**					
	(intercept)	−5.84	0.71	3.2 × 10^−16^	2.92 × 10^−3^
	age	0.00	0.02	0.99	1.00
	Sex_Male	−0.27	0.18	0.12	0.76
	TBL	0.02	0.00	2.0 × 10^−7^	1.02
	Sector_Cranial	2.72	0.87	1.8 × 10^−3^	15.17
	TBL:Sector_Cranial	−0.02	0.00	5.3 × 10^−4^	0.98
**(ii) zero-inflation model:**					
	(intercept)	5.61	2.14	8.8 × 10^−3^	272.62
	age	−0.21	0.11	0.05	0.81
	Sex_Male	−0.20	0.49	0.68	0.82
	TBL	−0.04	0.02	0.02	0.96
	Sector_Cranial	−0.37	2.26	0.87	0.69
	TBL:Sector_Cranial	0.01	0.02	0.60	1.01

**Figure 5.**
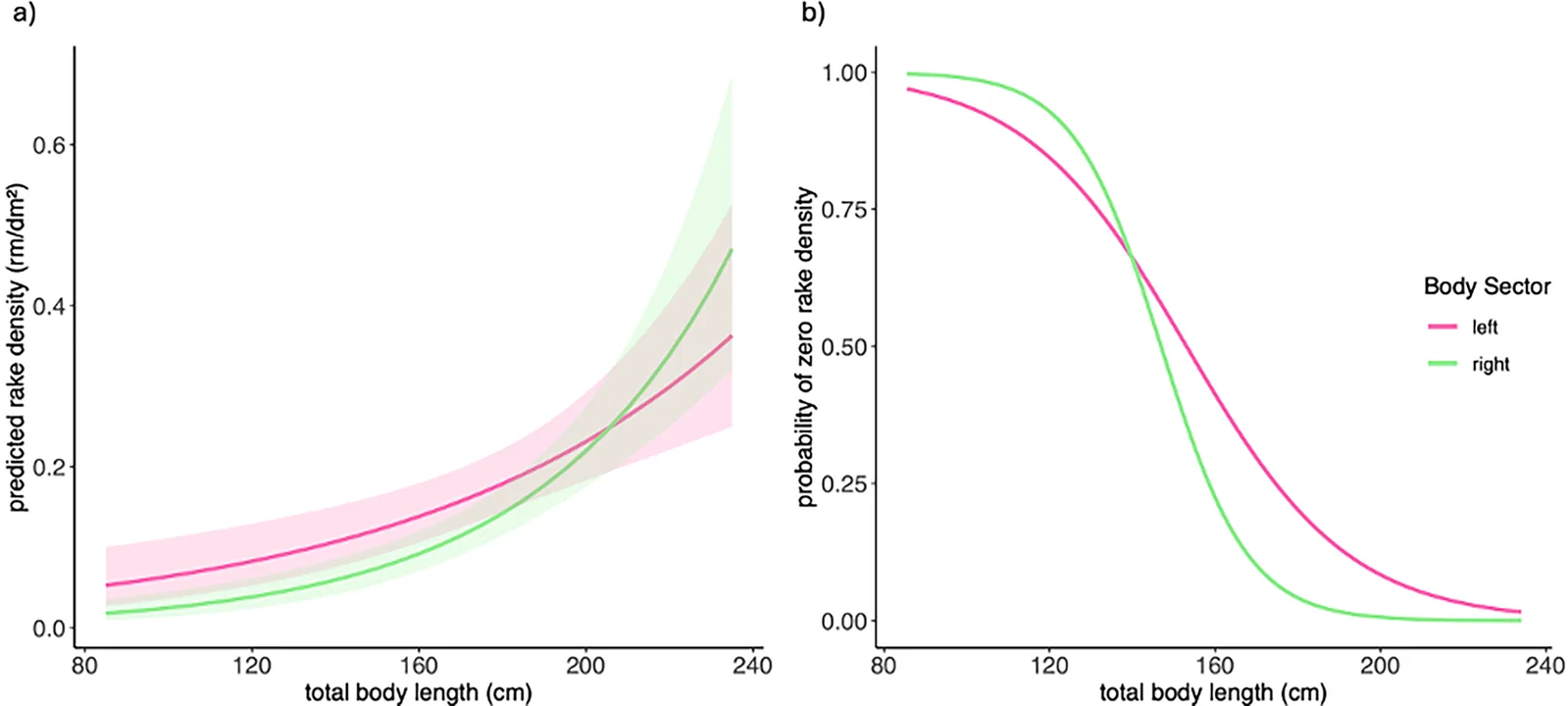
Predicted rake density and probability of zero rake density in relation to TBL across left and right body sectors in common dolphins (*Delphinus delphis*) assessed post-mortem, based on the best-fitting zero-inflated generalized linear mixed model (ZIGLMM). (a) Predicted positive rake density (rm dm^−2^) from the conditional component, shown for left (pink) and right (green) body sectors. (b) Predicted probability of zero rake density from the zero-inflation component, shown for the same sector groupings. Predictions account for the sector grouping × TBL interaction and include individual ID as a random effect. Lines represent model predictions, and shaded areas indicate 95% confidence intervals.

The zero-inflation component indicated that the probability of zero rake density also varied with body length and left- or right-hand side. Larger dolphins had a higher predicted probability of zero rake density on the right-hand side, while smaller individuals had a higher predicted probability of zero rake density on the left-hand side (*p* = 0.041; table 3a–ii, figure 5b). There was no evidence in this component that sex impacted the probability of having zero rake density (*p* = 0.88; table 3a–ii).

#### 3.3.2. Dorsum–ventrum

The best-fitting model for the dorsum and ventrum sectors included body sector grouping and body length as predictor variables, with an interaction effect between sector grouping and body length (supplementary material, table S8).

The conditional component revealed that rake density was higher on the dorsum compared to the ventrum, although this result was not statistically significant (*p* = 0.147; table 3b–i). Rake density increased by approximately 1.7% per cm increase in body length (*p* = 5.47×10⁻^9^; table 3b–i). The positive interaction term suggested that rake density may increase with body length at a greater rate on the ventrum compared to the dorsum (figure 6a), although this result is not significant (*p* = 0.135; table 3b–i).

**Figure 6.**
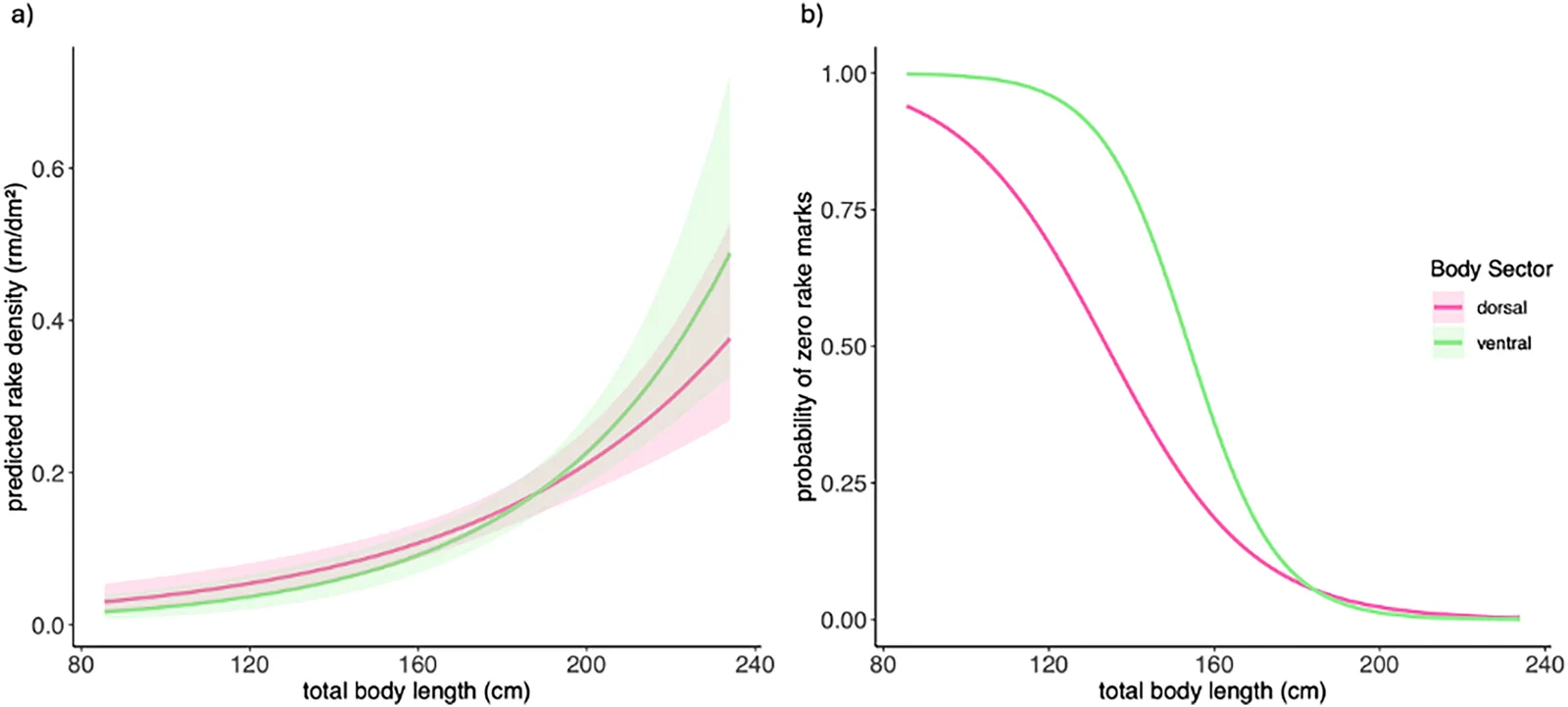
Predicted rake density and probability of zero rake density in relation to TBL across dorsal and ventral body sectors in common dolphins (*Delphinus delphis*) assessed post-mortem, based on the best-fitting zero-inflated generalized linear mixed model (ZIGLMM). (a) Predicted positive rake density (rm dm^−2^) from the conditional component, shown for dorsal (pink) and ventral (green) body sectors. (b) Predicted probability of zero rake density from the zero-inflation component, shown for the same sector groupings. Predictions account for the sector grouping × TBL interaction and include individual ID as a random effect. Lines represent model predictions, and shaded areas indicate 95% confidence intervals.

The zero-inflation component suggested a higher probability of zero rake density on the ventrum compared with the dorsum (*p* = 0.031; table 3b–ii). In addition, the odds of zero rake density decreased by 5.5% per cm increase in body length (*p* = 3.78 × 10⁻⁴; table 3b–ii). There was also weak evidence for an interaction between sector and body length (*p* = 0.059; table 3b–ii), suggesting that the probability of zero rake density declines at a greater rate with increasing body length on the ventrum than the dorsum (figure 6b).

#### 3.3.3. Cranial versus caudal

The best-fitting model for the cranial and caudal regions included sector grouping, age, sex and body length as predictor variables, with an interaction effect between sector grouping and body length (supplementary material, table S9).

The conditional component showed that larger common dolphins had higher caudal rake density relative to cranial, while smaller individuals showed relatively higher cranial rake density (*p* = 2.03 × 10⁻^7^ and *p* = 5.28 × 10⁻^4^, respectively; table 3c–i). Rake density was higher in the cranial sector at mean body length (*p* = 0.0018; table 3c–i), but shifted towards higher caudal rake density with increasing body length (figure 7a). Males showed slightly lower rake density than females, though this effect was not statistically significant (*p* = 0.12; table 3c–i). Age was not associated with rake density (*p* = 0.99; table 3c–i).

**Figure 7.**
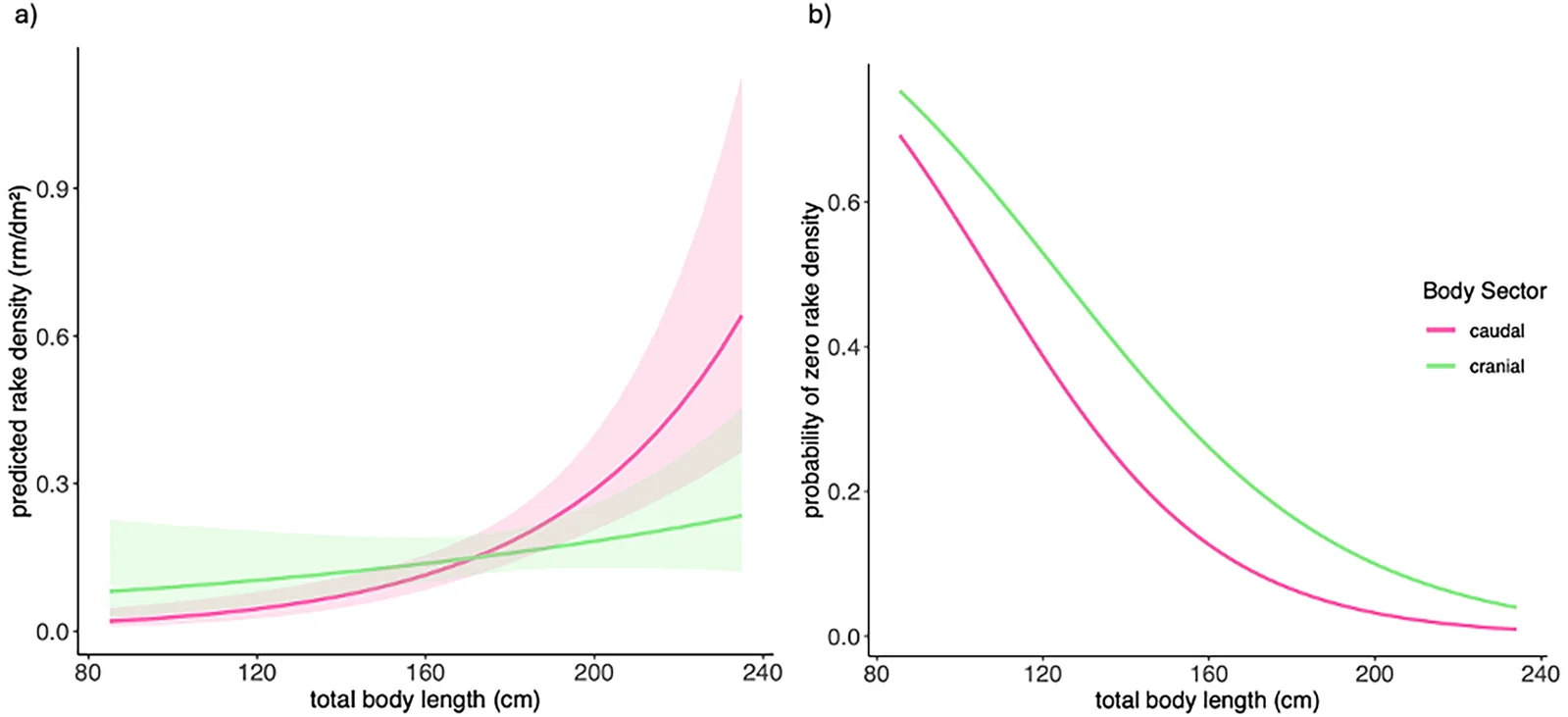
Predicted rake density and probability of zero rake density in relation to TBL across caudal and cranial body sectors in common dolphins (*Delphinus delphis*) assessed post-mortem, based on the best-fitting zero-inflated generalized linear mixed model (ZIGLMM). (a) Predicted positive rake density (rm dm^−2^) from the conditional component, shown for caudal (pink) and cranial (green) body sectors. (b) Predicted probability of zero rake density from the zero-inflation component, shown for the same sector groupings. Predictions account for the sector grouping × TBL interaction and include individual ID as a random effect. Lines represent model predictions, and shaded areas indicate 95% confidence intervals.

The zero-inflation component showed that larger individuals were less likely to have zero rake density (*p* = 0.018; table 3c–ii). Older individuals were slightly less likely to have zero rake density (*p* = 0.053; table 3c–ii), though this effect was not statistically significant. Females had a slightly higher estimated probability of zero rake density than males (*p* = 0.68; table 3c–ii), and the cranial body sector had a slightly lower estimated probability of zero rake density relative to caudal (*p* = 0.87; table 3c–ii). Neither of these results was statistically significant. The interaction between body sector and body length indicated a slightly weaker negative effect of body length on the probability of having zero rake marks in the cranial relative to caudal sector (figure 7b). However, this interaction was not statistically significant (*p* = 0.60; table 3c–ii).

## 4. Discussion

### 4.1. Occurrence and prevalence of rake marks

#### 4.1.1. Sex, age and body length

Male common dolphins were more likely to have at least one rake mark (higher occurrence) but had fewer rake marks overall across the body (lower prevalence) than females (table 1). This pattern of higher rake mark occurrence in males aligns with findings in other delphinid species, including male killer whales [7], Indo-Pacific and common bottlenose dolphins [16,17,40,68], as well as common dolphins off the coast of Ireland [69]. In contrast, studies that measure the extent of rake marks across the body (i.e. rake mark prevalence or rake density) often report higher rake density in males, including killer whales [7], common bottlenose dolphins [21], Amazon river dolphins (*Inia geoffrensis*) [51] and Indo-Pacific humpback dolphins (*Sousa chinensis*) [18].

Sex differences in social aggression vary widely across social mammals and are shaped by underlying social systems and reproductive strategies. For example, comparative analyses of primates show that male-biased dominance and frequent aggressive interactions are typically associated with polygynous, group-living systems, whereas females may experience equally or more intense competition owing to sexual coercion, philopatry or offspring investment [70]. Common dolphins form complex social groups with the potential for both male competition and female reproductive constraints; therefore, similar dynamics may contribute towards their social interactions [23,26].

One possible explanation could be that male common dolphins engage in more frequent but lower-intensity conflicts than females, resulting in higher rake mark occurrence but lower prevalence. Conversely, females may experience less frequent but more severe aggression, potentially linked to agonistic maternal behaviours such as calf protection, or responses to male harassment and coercion [14,21,71]. Alternatively, the results may be attributed to females experiencing repeated or prolonged instances of non-aggressive social behaviours, such as socio-sexual interactions or affiliative play. This could lead to a higher prevalence of rake marks. In contrast, males might engage in these behaviours more often, but in shorter bouts, leading to a higher occurrence but lower prevalence of rake marks.

Sex differences in healing rates may further influence measures of rake mark occurrence and prevalence. Cutaneous wounds heal faster in females than males in humans (*Homo sapiens*) [72–74], and mice (*Mus musculus*) [75]. A similar pattern has also been observed in Indo-Pacific bottlenose dolphins, possibly owing to both immune response and variation in the severity of aggression received [17]. If female common dolphins also heal more quickly, their rake mark occurrence would appear lower, making social interactions less detectable even if they occur as frequently as in males, while the higher prevalence of rake marks could reflect the prolonged healing of more severe wounds [16,17].

We found no relationship between age and rake mark prevalence or occurrence in common dolphins (table 1). However, social interactions are known to vary with age in a range of social mammals, and such sociality can shift across the lifespan owing to a combination of senescence, adaptive behavioural changes and demographic processes [76]. For example, for Barbary macaques (*Macaca sylvanus*), this can result in reduced social network size or interaction frequency in older individuals [77]. Alternatively, in some cases, stable or even increased social engagement can occur with age, as seen in rhesus macaques (*M. mulatta*) [76]. This indicates that age can have a variable effect on social interactions. For several cetaceans, age-related variation in social interactions has been well-documented (e.g. beluga whales, *Delphinapterus leucas* [78]), with links to rake marks Indo-Pacific and common bottlenose dolphins [16,17], killer whales [7] and Indo-Pacific humpback dolphins [18]. This is probably due to behavioural changes over their lifespan. One explanation for the lack of an observed age relationship in common dolphins could be that similar levels of social interactions resulting in rake marks occur across age classes. Alternatively, age-related differences in rake mark healing might affect the detectability of rake marks. In several mammals, including humans, wound healing rates decline with age owing to factors like poor body condition and reduced immune function [79–81]. Should older dolphins heal more slowly, rake marks might persist longer, masking age-related patterns of social interactions.

In contrast, rake mark occurrence and prevalence increased marginally with body length in common dolphins (table 1) [69]. This could be explained by larger individuals engaging in more frequent and prolonged social interactions, or more simply, that greater surface area increases the likelihood of being raked. Body size, among other physical characteristics, can act as a signal of social status in wild animals, influencing their aggressive interactions [49]. It also plays an important role in fighting ability, and often dictates the likelihood and outcome of aggressive interactions, correlating with dominance rank and reproductive success in a broad range of taxa [49,82,83].

Body size correlates with reproductive success in killer whales, possibly owing to advantages in aggressive contests [84]. For male common dolphins, the large testes-to-body size ratio favours sperm competition over physical contests, indicating that post-anal hump size may act as a signal of dominance [44,60,85,86]. Thus, larger dolphins may accrue more rake marks owing to aggressive interactions linked to dominance relationships. Therefore, larger and potentially more dominant male common dolphins may be more prone to engage in frequent aggressive interactions owing to their physical advantage, leading to increased rake mark occurrence. In contrast, those that are smaller may be more likely to avoid high-risk encounters with conspecifics, resulting in fewer rake marks [17,18,49,84].

It is also possible that larger dolphins may be able to withstand or sustain affiliative social interactions for longer durations owing to their relative energetic capacity, resulting in a higher prevalence of rake marks. Larger toothed cetacean (odontocete) species exhibit greater social complexity, suggesting a potential link between size and the capacity for extended social engagement, although this link has yet to be explored within species [87]. However, the increased surface area in larger individuals may be a more likely explanation for the relationship between rake occurrence and body length, as smaller common dolphins may be more likely to evade aggression from their larger counterparts.

#### 4.1.2. Body condition

Body condition had no effect on rake mark occurrence or prevalence (table 1). Considered as a proxy for health through nutritional index [88–91], body condition has been linked to fighting ability in some species, with healthier individuals demonstrating enhanced energy, strength and agility that may influence dominance and contest outcomes [83]. However, our findings suggest that body condition does not significantly affect the frequency or severity of social interactions between common dolphins. Another possibility is that healing rates may vary with body condition. Dolphins in poor health might experience delayed healing, leading to longer-lasting rake marks and greater detectability, as noted in other mammals, including humans [92,93]. This could mask a potential relationship between body condition and social interactions, as variations in healing rates might confound scarring patterns as a measure of behaviour, although this is yet to be explored within this species.

#### 4.1.3. Sexual maturity and reproductive status

We detected no relationship between sexual maturity and rake mark occurrence or prevalence (table 1). This suggests that rake marks are not solely associated with reproductive behaviours such as sexual coercion of females and male competition for dominance and reproduction, as suggested for many delphinids [16–18,21] including Indo-Pacific and common bottlenose dolphins [16,17,21]. Sexual coercion, including forced mating and persistent harassment, is widely documented across mammals and is often associated with increased aggression, particularly by males towards females [94–96]. For example, males in some primate species engage in repeated harassment to increase mating success [97,98], while male bottlenose dolphins use physical force and form alliances to control and mate with females [14,21,99,100]. These behaviours are thought to shape social interactions across a range of taxa and life-history stages, including chimpanzees (*Pan troglodytes*; [101]) and mandrills (*Mandrillus sphinx*; [102]), and would therefore be expected to contribute to rake mark patterns in common dolphins.

However, the absence of a relationship with sexual maturity in this study suggests that such behaviours may not be restricted to sexually mature individuals, or that individuals across life stages experience comparable levels of social interaction that result in rake marks. Instead, rake marks probably arise in other contexts as well, including affiliative play or socio-sexual behaviour [35,50]. This aligns with Scott *et al.* [16], who found rake marks to be common in juveniles, indicating that they occur in a variety of behavioural contexts.

Female rake mark occurrence and prevalence showed some variation with reproductive status (table 2). However, interpretation of results for resting mature females should be treated cautiously, as all individuals within this category exhibited non-zero rake densities, limiting model interpretation. Despite this limitation, immature females exhibited a higher rake mark occurrence and prevalence than pregnant and/or lactating females. Resting mature females may experience heightened social interactions, including affiliative and socio-sexual behaviour and/or aggression owing to their availability for reproduction, aligning with hypotheses of increased sexual coercion by males [16,21]. For example, Scott *et al.* [16] observed that cycling Indo-Pacific bottlenose dolphin females showed new rake marks more frequently than non-cycling females, probably owing to male coercion. Pregnant and/or lactating females exhibited the lowest rake mark occurrence and prevalence, potentially reflecting reduced male aggression owing to their reproductive unavailability [16,45,103] or their tendency to avoid social interactions during calf rearing [71,104].

Reproductive status is also known to influence wound healing across mammals. In primates, lactating females heal more slowly than those that are pregnant or cycling, probably owing to energy trade-offs between reproduction and immune function [105,106]. However, this pattern does not explain our findings, as pregnant and/or lactating common dolphins had the lowest rake mark occurrence and prevalence, more probably reflecting reduced aggression or affiliative behaviour towards these individuals rather than faster healing rates (table 2). Note that, owing to sample size, we did not distinguish between pregnant, lactating or pregnant and lactating females, as wound healing may vary within this subgroup. Nevertheless, the observed variation in rake marks by reproductive status appears more closely related to social interactions rather than differences in wound healing.

### 4.2. Scar position

The distribution of lesions, including rake marks, across the body has been examined in various delphinid species, including Indo-Pacific humpback dolphins [18], Indo-pacific bottlenose dolphins [15,16] and common dolphins [53,69]. Patterns vary across species, and study contexts, particularly between live and post-mortem examinations, where ventral surfaces are often obscured in live animals. Among these, Indo-Pacific bottlenose dolphins show more rake marks on the posterior peduncle [16], Indo-Pacific humpback dolphins on the flank [18] and common dolphins in Irish waters on the torso [69]. In New Zealand, live common dolphins most frequently show lesions on the anterior peduncle and dorsal fin [53].

Dorsal rake marks may indicate that this body region is presented defensively during aggressive interactions [18]. Higher rake density observed on the dorsum compared to the ventrum suggests variation in the amount that each sector is displayed across social contexts (table 3b–i). This could possibly reflect frequent defensive posturing during forceful mating attempts, competition for resources or socio-sexual and play behaviour. Rake marks on the ventrum may be indicative of infrequent socio-sexual or mating interactions where the ventrum is exposed [107,108].

Smaller dolphins displayed more cranial rake marks, potentially from head-to-head contests during competitive interactions over resources or mates [52] (table 3c–i). Larger dolphins exhibited more caudal rake marks, suggesting a tendency to adopt defensive strategies typical of less competitive individuals. Caudal scarring might also result from tail-slapping of the recipient in retaliation towards the instigator [109]. However, the behavioural context remains uncertain.

Some evidence of behavioural lateralization was observed (table 3a). Smaller dolphins showed marginally higher right-sided rake mark occurrence, and left-sided prevalence of rake marks, while larger individuals showed the opposite pattern. This could possibly be related to known lateralization behaviours, such as left-side rubbing [110], calves swimming on their mothers’ right-side [111] and right-side foraging [112]. Such trends may change with increased age and independence, as observed in other cetaceans [111]. These results warrant further research to investigate the potential drivers of such patterns.

### 4.3. Abiotic factors

Temporal variation may also influence the social behaviour of common dolphins. In female common dolphins in New Zealand waters, weak reproductive seasonality has been observed [57], suggesting that reproductive activity occurs throughout the year but may still fluctuate in intensity over time. Temporal changes in primary productivity are also known to influence common dolphin diet in New Zealand [113,114], and may therefore indirectly affect social behaviour by altering resource availability. In addition, common dolphins exhibit fission–fusion social dynamics, where group composition and behaviour vary over time, with behavioural states shown to change across months [23]. These changes would probably be reflected in the occurrence and prevalence of rake marks. However, such temporal effects were not modelled in this study, as stranding time and location may not accurately represent the timing or location of the social interactions during which rake marks were acquired.

In addition to these influences on social behaviour, abiotic factors may influence wound healing rates, affecting rake mark longevity and the likelihood of their detection as evidence of social interactions [115–119]. Hypo-salinity and low water temperature correlate with higher prevalence of epidermal lesions [115,116,117,120,121] and slower wound healing in delphinids [118,120,122]. Low salinity increases water absorption in the epidermis, causing freshwater skin lesions [116,120,123]. Similarly, low temperatures have been linked to increased expression of tattoo lesions, which may resolve as water temperatures rise, probably owing to improved viral defenses [115,121,124].

As scar longevity can affect the prevalence, accumulation and likelihood of rake mark detection over time, abiotic factors such as salinity and sea-surface temperature represent uncontrolled variables that may influence the observed rake marks on common dolphins. This should be considered alongside any inference of social interactions.

### 4.4. Study limitations

Temporal variation was not incorporated into this study, as stranding records may not accurately reflect the timing or context of the social interactions during which rake marks were acquired. However, such factors may influence both environmental conditions and social behaviour, which could result in unexplained variation in the dataset. Longitudinal studies using photographic identification techniques that allow them to follow known individuals over time while incorporating concurrent abiotic conditions would be better able to detect how these factors influence the occurrence and detectability of rake marks.

While recognizing the potential effects of abiotic factors on social behaviour and rake mark persistence and detectability, we also acknowledge there are wider limitations to consider. For example, post-mortem sampling can introduce biases owing to injury and potential overrepresentation of unhealthy individuals. In addition, the level of decomposition of the carcass and thus rake mark detectability could also vary depending on the cause of death. Where possible, we mitigated these concerns by (i) controlling for the decomposition state of animals included in this study, (ii) accounting for loss of skin from both external injuries and areas of post-mortem skin loss by assessment of rake mark as a percentage of visible skin, and (iii) inclusion of incidentally bycaught and mass stranding animals in which disease was not an underlying cause of death.

Finally, while rake marks are an indirect measure of social interactions, we acknowledge that this is still an underestimate of interactions given that not all encounters result in visible scars.

### 4.5. Conclusion

Rake mark occurrence and prevalence varied with sex and body length in common dolphins examined post-mortem from New Zealand. Age, sexual maturity, body condition, and female reproductive status were not strong predictors of rake mark occurrence or prevalence. While it remains unclear in what context scarring occurs, and whether it is more attributable to aggressive versus affiliative social interactions, rake mark positioning indicates behavioural strategies spanning a range of social contexts. Accordingly, this study offers preliminary insights into the relationship between rake marks and social interactions, enhancing our understanding of the sociobiology of common dolphins, as well as potentially providing insights to the social behaviour of non-marine group-living mammals. Future research would benefit from longitudinal photo-identification datasets that track known individuals over time, enabling a direct connection to be drawn between social behaviour, life-history stage and rake mark acquisition. Incorporating fine-scale behavioural observations and social network analyses would further help to distinguish the relative contributions of aggression, sexual coercion and affiliative interactions towards rake marks. In addition, accounting for temporal variation in environmental conditions and incorporating wound healing rates could improve interpretation of scarring patterns as a measure of social interactions.

## Supplementary Material



## Data Availability

Data and relevant code for this research work are stored in GitHub [https://github.com/Marine-Vertebrate-Ecology/Crisp-et-al-rake-marks-Delphinus-delphis] and have been archived within the Zenodo repository [125]. Supplementary material is available online [126].
