# Supplementary material for: Ontogeny of rake mark scarring on common dolphins (Delphinus delphis): insights to aggression and affiliation?
Source: R Soc Open Sci. 2026 Oct 14;13(10):252175. doi: 10.1098/rsos.252175 (PMC13682422; doi:10.1098/rsos.252175)
Supplement: Supplementary file 1 [file rsos.252175.zip › rsos.252175/rsos252175_si_001.docx]

**SUPPLEMENTARY MATERIALS**

**Ontogeny of rake mark scarring on common dolphins (*Delphinus delphis*): insights to aggression and affiliation?**

Hannah R. Crisp^1^, Katharina J. Peters^2,3^, Emily I. Palmer^1^, Frédérik Saltré^4,5^, Emma L. Betty^1^, Eva-Maria F. Hanninger^1^, Karen A. Stockin^1^

^1^Cetacean Ecology Research Group, School of Natural Sciences, Massey University, Auckland, New Zealand

^2^Marine Vertebrate Ecology Lab, Environmental Futures, School of Science, University of Wollongong, Wollongong, Australia

^3^ Securing Antarctica’s Environmental Future, University of Wollongong, Wollongong, New South Wales, 2522, Australia

^4^Biogeography Ecology & Modelling, School of Life Sciences, University Technology Sydney, Sydney, New South Wales 2007, Australia

^5^Australian Museum Research Institute, Australian Museum, Sydney, New South Wales 2010, Australia

**TABLES**

**Table S1** Table used to record body morphometrics for common dolphin (*Delphinus delphis*) cadavers from 2006 to 2024 in New Zealand.

| 1. Total body length | cm | 2. Snout to origin pectoral fin | cm |
| --- | --- | --- | --- |
| 3. Snout to origin dorsal fin | cm | 4. Snout to tip dorsal fin | cm |
| 5. Eye to blowhole | cm | 6. Dorsal fin height | cm |
| 7. Dorsal fin lateral base | cm | 8. Fluke width | cm |
| 9. Fluke length | cm | 10. Internal pectoral fin length | cm |
| 11. External pectoral fin length | cm | 12. Pectoral fin width | cm |
| 13. Girth at pectoral fin | cm | 14. Girth at navel | cm |

**Table S2** Characteristics of each body condition score for common dolphin (Delphinus delphis) cadavers from 2006 to 2024 in New Zealand.

| Score | Definition |
| --- | --- |
| 1. Good | Rounded caudal and cranial dorsal flanks. |
| 2. Moderate | Sloping caudal and cranial dorsal flanks. |
| 3. Poor | Hollow caudal and cranial dorsal flanks. The transverse process of the lumbar vertebrae, and a dorsal indentation caudal to the head are visible. |

**Table S3** Characteristics of each decomposition stage.

| Score | Definition |
| --- | --- |
| 1. Fresh | Dead for less than 48 hours, no signs of rigor mortis, clear cornea, eyes are firm (i.e. not flaccid). |
| 2. Mild | Rigor mortis; early decomposition taking place, e.g., surface decay of eyes and skin; no decomposition smell; organs intact. |
| 3. Moderate | Peeling of skin visible; decomposition signs visible, although moderate, e.g., a change in skin and organ colour or consistency may be seen; moderate decomposition odor present. |
| 4. Severe decomposition | Collapsing carcass; skin sloughing; strong decomposition smell; soft blubber, with oil or gas pockets possibly present; muscle easily torn, possibly coming away from bones; thin and black blood; organs may be torn or difficult to detect; gas in gut. |

**Table S4** Summary of rake density in common dolphins (*Delphinus delphis*) by sex, sexual maturity, body condition, and female reproductive status, showing the observed rake density range, mean ± standard deviation (SD), and sample size (n).

| Category | Subgroup | Rake density range (dm^2^) | Mean ± SD | n |
| --- | --- | --- | --- | --- |
| Sex | Females | 0 – 1.06 | 0.20 ± 0.23 | 56 |
|  | Males | 0 – 0.60 | 0.13 ± 0.15 | 46 |
| Sexual maturity | Immature (all) | 0 – 0.74 | 0.08 ± 0.15 | 56 |
|  | Mature (all) | 0 – 1.06 | 0.28 ± 0.20 | 46 |
|  | Immature females | 0 – 0.74 | 0.09 ± 0.18 | 27 |
|  | Mature females | 0 – 1.06 | 0.31 ± 0.22 | 29 |
|  | Immature males | 0 – 0.39 | 0.08 ± 0.12 | 29 |
|  | Mature males | 0.03 – 0.60 | 0.22 ± 0.15 | 17 |
| Body condition | Good | 0 – 0.74 | 0.17 ± 0.19 | 66 |
|  | Moderate | 0 – 1.06 | 0.17 ± 0.22 | 31 |
|  | Poor | 0 – 0.285 | 0.09 ± 0.12 | 5 |
| Female reproductive status | Immature | 0 – 0.74 | 0.09 ± 0.18 | 27 |
|  | Pregnant and/or lactating | 0 – 0.65 | 0.28 ± 0.17 | 23 |
|  | Resting mature | 0.04 – 1.06 | 0.41 ± 0.35 | 6 |

**Table S5** Performance values for zero-inflated generalised linear models (ZIGLMs). These models incorporate sex (male or female), maturity (sexual maturity; immature or mature), age (years), TBL (total body length, cm), and BC (body condition; good, moderate, or poor) to estimate the rake density (rm/dm2) of each individual. Note: k = number of parameters, LogLik = minimized negative log-likelihood, AICc = corrected Akaike Information Criterion for small sample sizes, wAICc = Akaike weight adjusted to sum up to 1, ER = evidence ratio, calculated in comparison to the best-performing model.

| Model | k | ER | AICc | wAICc | LogLik |
| --- | --- | --- | --- | --- | --- |
| Rake density (entire body) |  |  |  |  |  |
| ~ Sex + TBL | 7 | - | -65.17 | 0.55 | 40.19 |
| ~ Age + Sex + TBL + Maturity | 11 | 0.09 | -60.30 | 0.05 | 42.63 |
| ~ Sex + TBL + BC | 9 | 0.13 | -61.10 | 0.07 | 40.54 |
| ~ Age + Sex + TBL | 9 | 0.12 | -61.01 | 0.07 | 40.49 |
| ~ TBL | 5 | 0.22 | -62.10 | 0.12 | 36.37 |
| ~ Sex + TBL + Maturity | 9 | 0.11 | -60.75 | 0.06 | 40.36 |
| ~ Age + Sex + TBL + B | 11 | 0.02 | -56.88 | 0.01 | 40.92 |
| ~ TBL + BC | 7 | 0.01 | -55.53 | 0.00 | 42.86 |
| ~ Age + TBL + Maturity | 9 | 0.03 | -58.31 | 0.02 | 36.75 |
| ~ TBL + Maturity + BC + Sex | 11 | 0.01 | -56.31 | 0.01 | 40.64 |
| ~ Age + TBL | 7 | 0.03 | -57.94 | 0.01 | 36.57 |
| ~ TBL + Maturity | 7 | 0.03 | -57.89 | 0.01 | 36.55 |
| ~ Age + TBL + Maturity + BC + Sex | 13 | 0.02 | -57.11 | 0.01 | 38.54 |
| ~ Age + TBL + BC | 9 | 3.71x10^-3^ | -53.97 | 2.04x10^-3^ | 36.98 |
| ~ TBL + Maturity + BC | 9 | 3.21x10^-3^ | -53.69 | 1.77x10^-3^ | 36.83 |
| ~ Age + BC + TBL + Maturity | 11 | 1.94x10^-3^ | -52.67 | 1.07x10^-3^ | 38.82 |
| ~ Age + Sex + Maturity | 9 | 4.49x10^-6^ | -40.54 | 2.47x10^-6^ | 30.26 |
| ~ Age + Maturity | 7 | 5.03x10^-6^ | -40.77 | 2.77x10^-6^ | 27.99 |
| ~ Age + Sex + BC + Maturity | 11 | 8.40x10^-7^ | -37.19 | 4.62x10^-7^ | 31.08 |
| ~ Age + BC + Maturity | 9 | 9.16x10^-7^ | -37.36 | 5.04x10^-7^ | 28.67 |
| ~ Age | 5 | 9.34x10^-7^ | -37.40 | 5.14x10^-7^ | 24.02 |
| ~ Age + Sex | 7 | 6.13x10^-7^ | -36.55 | 3.37x10^-7^ | 25.88 |
| ~ Age + Sex + BC | 9 | 1.54x10^-7^ | -33.80 | 8.50x10^-8^ | 26.89 |
| ~ Maturity | 5 | 1.24x10^-12^ | -10.33 | 6.80x10^-13^ | 10.48 |
| ~ Maturity + BC | 7 | 5.65x10^-13^ | -8.76 | 3.11x10^-13^ | 11.98 |
| ~ Sex + Maturity | 7 | 2.81x10^-13^ | -7.36 | 1.54x10^-13^ | 11.28 |
| ~ Sex + Maturity + BC | 9 | 1.37x10^-13^ | -5.93 | 7.54x10^-14^ | 12.95 |
| ~ Sex | 5 | 1.80x10^-19^ | 21.15 | 9.91x10^-20^ | -5.26 |
| ~ BC | 5 | 7.54x10^-20^ | 22.90 | 4.15x10^-20^ | -6.13 |
| ~ Sex + BC | 7 | 3.80x10^-20^ | 24.27 | 2.09x10^-20^ | -4.53 |

**Table S6** Performance values for zero-inflated generalised linear models (ZIGLMs) analysing rake density of females for the entire body surface in common dolphins (Delphinus delphis) assessed postmortem. These models incorporate reproductive status (immature, pregnant and/or lactating, and resting mature), age (years), TBL (total body length, cm), and BC (body condition; good, moderate, or poor) to estimate the rake density (rm/dm^2^) of each individual. Note: k = number of parameters, LogLik = minimized negative log-likelihood, AICc = corrected Akaike Information Criterion for small sample sizes, wAICc = Akaike weight adjusted to sum up to 1, ER = evidence ratio, calculated in comparison to the best-performing model.

| Model | k | ER | AICc | wAICc | LogLik |
| --- | --- | --- | --- | --- | --- |
| Female rake density (entire body) |  |  |  |  |  |
| ~ Reproductive_status + TBL | 4 | - | -5.79 | 0.57 | 13.98 |
| ~ Reproductive_status + TBL + Age | 5 | 1.99 | -4.41 | 0.29 | 16.27 |
| ~ Reproductive_status + TBL + BC | 5 | 6.46 | -2.06 | 0.09 | 15.10 |
| ~ Reproductive_status + TBL + BC + Age | 13 | 12.01 | -0.82 | 0.05 | 17.85 |
| ~ Reproductive_status + Age | 4 | 1.54x10^2^ | 4.28 | 3.72x10^-3^ | 8.86 |
| ~ Reproductive_status + BC + Age | 5 | 1.43 x10^3^ | 8.75 | 4.00x10^-4^ | 9.70 |
| ~ Reproductive_status | 3 | 4.63x10^4^ | 15.70 | 1.24x10^-5^ | 0.34 |
| ~ Reproductive_status + BC | 4 | 2.33x10^5^ | 18.93 | 2.46x10^-6^ | 1.54 |

**Table S7** Performance values for zero-inflated generalised linear mixed models (ZIGLMMs) analysing left versus right sector groupings in common dolphins (Delphinus delphis) assessed postmortem. These models incorporate sex (male or female), sector (sector grouping; left or right), maturity (sexual maturity; immature or mature), age (years), TBL (total body length, cm), BC (body condition; good, moderate, or poor), and ID (individual identity) to estimate the rake density (rm/dm^2^) of each individual. Note: k = number of parameters, LogLik = minimized negative log-likelihood, AICc = corrected Akaike Information Criterion for small sample sizes, wAICc = Akaike weight adjusted to sum up to 1, ER = evidence ratio, calculated in comparison to the best-performing model.

| Model | k | ER | AICc | wAICc | LogLik |
| --- | --- | --- | --- | --- | --- |
| Rake density (left vs right) |  |  |  |  |  |
| ~ Sex + Sector * TBL + (1 \| ID) | 13 | - | -84.22 | 0.97 | 56.08 |
| ~ Sex + TBL + Sector + (1 \| ID) | 11 | 1.27x10^-3^ | -75.43 | 0.01 | 49.41 |
| ~ Sector + TBL + (1 \| ID) | 9 | 1.20x10^-4^ | -75.16 | 0.01 | 47.05 |
| ~ Sex + Age + TBL + Sector + (1 \| ID) | 13 | 1.43x10^-3^ | -71.12 | 1.39x10^-3^ | 49.53 |
| ~ Maturity + TBL + Sector + (1 \| ID) | 11 | 1.39x10^-4^ | -71.00 | 1.31x10^-3^ | 47.20 |
| ~ Age + TBL + Sector + (1 \| ID) | 11 | 1.38x10^-4^ | -70.98 | 1.30x10^-3^ | 47.19 |
| ~ Sex + Maturity + TBL + Sector + (1 \| ID) | 13 | 1.31x10^-3^ | -70.95 | 1.27x10^-3^ | 49.44 |
| ~ BC + Sex + TBL + Sector + (1 \| ID) | 15 | 8.12x10^-3^ | -69.95 | 7.73x10^-3^ | 51.26 |
| ~ BC + TBL + Sector + (1 \| ID) | 13 | 4.78x10^-4^ | -68.93 | 4.64x10^-4^ | 48.43 |
| ~ Age + Maturity + TBL + Sector + (1 \| ID) | 13 | 1.55x10^-4^ | -66.67 | 1.50x10^-4^ | 47.30 |
| ~ Sex + Maturity + TBL + Age + Sector + (1 \| ID) | 15 | 1.46x10^-3^ | -66.52 | 1.39x10^-4^ | 49.55 |
| ~ Sex + Age + BC + TBL + Sector + (1 \| ID) | 17 | 9.26x10^-3^ | -65.46 | 8.22x10^-5^ | 51.39 |
| ~ Maturity + BC + Sex + TBL + Sector + (1 \| ID) | 17 | 8.49x10^-3^ | -65.29 | 7.53x10^-5^ | 51.31 |
| ~ BC + Maturity + TBL + Sector + (1 \| ID) | 15 | 5.63x10^-4^ | -64.61 | 5.36x10^-5^ | 48.59 |
| ~ Age + TBL + BC + Sector + (1 \| ID) | 15 | 5.58x10^-4^ | -64.59 | 5.31x10^-5^ | 48.59 |
| ~ Sex + Age + Maturity + BC + TBL + Sector + (1 \| ID) | 19 | 9.55x10^-3^ | -60.67 | 7.50x10^-6^ | 51.43 |
| ~ BC + Age + Maturity + TBL + Sector + (1 \| ID) | 17 | 6.34x10^-4^ | -60.10 | 5.63x10^-6^ | 48.71 |
| ~ Maturity + Age + BC + Sector + (1 \| ID) | 14 | 1.22x10^-6^ | -54.68 | 3.74x10^-7^ | 42.46 |
| ~ Age + Sector + (1 \| ID) | 9 | 6.05x10^-12^ | -41.55 | 5.29x10^-10^ | 30.25 |
| ~ Age + Maturity + Sector + (1 \| ID) | 11 | 3.11x10^-11^ | -40.38 | 2.93x10^-10^ | 31.88 |
| ~ Age + Sex + Sector + (1 \| ID) | 11 | 1.47x10^-11^ | -38.88 | 1.39x10^-10^ | 31.13 |
| ~ Sex + Age + Maturity + Sector + (1 \| ID) | 13 | 5.57x10^-11^ | -37.00 | 5.41x10^-11^ | 32.47 |
| ~ Age + BC + Sector + (1 \| ID) | 13 | 4.42x10^-11^ | -36.53 | 4.29x10^-11^ | 32.23 |
| ~ Sex + Age + BC + Sector + (1 \| ID) | 15 | 1.41x10^-10^ | -34.21 | 1.35x10^-11^ | 33.40 |
| ~ Sector + Maturity + (1 \| ID) | 17 | 5.57x10^-10^ | -32.21 | 4.94x10^-12^ | 34.77 |
| ~ Sex + Age + Maturity + BC + Sector + (1 \| ID) | 9 | 3.40x10^-15^ | -26.59 | 2.97x10^-13^ | 22.76 |
| ~ Sex + Maturity + Sector + (1 \| ID) | 11 | 5.10x10^-15^ | -22.94 | 4.81x10^-14^ | 23.17 |
| ~ BC + Maturity + Sector + (1 \| ID) | 13 | 2.46x10^-14^ | -21.55 | 2.39x10^-14^ | 24.74 |
| ~ Sex + Maturity + BC + Sector + (1 \| ID) | 15 | 4.27x10^-14^ | -18.00 | 4.07x10^-15^ | 25.29 |
| ~ Sector + (1 \| ID) | 7 | 3.21x10^-29^ | 33.64 | 2.48x10^-26^ | -9.53 |
| ~ Sector + Sex + (1 \| ID) | 9 | 1.25x10^-28^ | 35.28 | 1.09x10^-26^ | -8.17 |
| ~ BC + Sector + (1 \| ID) | 11 | 3.78x10^-28^ | 37.52 | 3.57x10^-27^ | -7.07 |
| ~ BC + Sex + Sector + (1 \| ID) | 13 | 1.56x10^-27^ | 39.23 | 1.51x10^-27^ | -5.65 |

**Table S8** Performance values for zero-inflated generalised linear mixed models (ZIGLMMs) analysing dorsal versus ventral sector groupings in common dolphins (Delphinus delphis) assessed postmortem. These models incorporate sex (male or female), sector (sector grouping; dorsal or ventral), maturity (sexual maturity; immature or mature), age (years), TBL (total body length, cm), BC (body condition; good, moderate, or poor), and ID (individual identity) to estimate the rake density (rm/dm^2^) of each individual. Note: k = number of parameters, LogLik = minimized negative log-likelihood, AICc = corrected Akaike Information Criterion for small sample sizes, wAICc = Akaike weight adjusted to sum up to 1, ER = evidence ratio, calculated in comparison to the best-performing model.

| Model | k | ER | AICc | wAICc | LogLik |
| --- | --- | --- | --- | --- | --- |
| Rake density (dorsal vs ventral) |  |  |  |  |  |
| ~ Sector * TBL + (1 \| ID) | 11 | - | -76.84 | 0.52 | 50.11 |
| ~ Sector + TBL + (1 \| ID) | 9 | 0.38 | -74.89 | 0.20 | 46.91 |
| ~ Sex + TBL + Sector + (1 \| ID) | 11 | 0.20 | -73.66 | 0.11 | 48.52 |
| ~ Maturity + TBL + Sector + (1 \| ID) | 11 | 0.10 | -72.20 | 0.05 | 47.79 |
| ~ TBL + BC + Sector + (1 \| ID) | 11 | 0.05 | -71.00 | 0.03 | 47.19 |
| ~ Age + TBL + Sector + (1 \| ID) | 11 | 0.05 | -70.92 | 0.03 | 47.15 |
| ~ Sex + Maturity + TBL + Sector + (1 \| ID) | 13 | 0.04 | -70.13 | 0.02 | 49.03 |
| ~ Sex + BC + TBL + Sector + (1 \| ID) | 13 | 0.03 | -69.58 | 0.01 | 48.76 |
| ~ Age + Sex + TBL + Sector + (1 \| ID) | 13 | 0.02 | -69.34 | 0.01 | 48.64 |
| ~ TBL + Maturity + BC + Sector + (1 \| ID) | 13 | 0.01 | -68.26 | 7.19x10^-3^ | 48.10 |
| ~ Maturity + Age + TBL + Sector + (1 \| ID) | 13 | 0.01 | -67.68 | 5.37x10^-3^ | 47.81 |
| ~ Age + BC + TBL + Sector + (1 \| ID) | 13 | 7.10x10^-3^ | -66.94 | 3.71x10^-3^ | 47.44 |
| ~ Sex + Maturity + TBL + BC + Sector + (1 \| ID) | 15 | 4.34x10^-3^ | -65.95 | 2.26x10^-3^ | 49.27 |
| ~ Age + TBL + Sex + Maturity + Sector + (1 \| ID) | 15 | 3.49x10^-3^ | -65.52 | 1.82x10^-3^ | 49.05 |
| ~ BC + Age + Sex + TBL + Sector + (1 \| ID) | 15 | 2.91x10^-3^ | -65.16 | 1.52x10^-3^ | 48.87 |
| ~ Sex + Age + Maturity + BC + Sector + (1 \| ID) | 15 | 1.37x10^-3^ | -63.64 | 7.13x10^-4^ | 48.11 |
| ~ BC + Sex + Age + Maturity + TBL + Sector + (1 \| ID) | 17 | 4.10x10^-4^ | -61.24 | 2.14x10^-4^ | 49.28 |
| ~ Maturity + Age + BC + Sector + (1 \| ID) | 13 | 7.47x10^-5^ | -57.83 | 3.90x10^-5^ | 42.88 |
| ~ Age + Maturity + Sector + (1 \| ID) | 11 | 2.35x10^-9^ | -37.10 | 1.23x10^-9^ | 30.24 |
| ~ Age + Sector + (1 \| ID) | 9 | 1.07x10^-9^ | -35.53 | 5.60x10^-10^ | 27.23 |
| ~ Age + Sex + Maturity + Sector + (1 \| ID) | 13 | 3.69x10^-10^ | -33.40 | 1.93x10^-10^ | 30.67 |
| ~ Sex + Age + Sector + (1 \| ID) | 11 | 2.23x10^-10^ | -32.39 | 1.17x10^-10^ | 27.89 |
| ~ Sector + Age + BC + (1 \| ID) | 11 | 1.29x10^-10^ | -31.29 | 6.74x10^-10^ | 27.34 |
| ~ Sex + Age + BC + Maturity + Sector + (1 \| ID) | 15 | 4.65x10^-11^ | -29.25 | 2.43x10^-11^ | 30.92 |
| ~ Age + BC + Sex + Sector + (1 \| ID) | 13 | 2.78x10^-11^ | -28.23 | 1.45x10^-11^ | 28.08 |
| ~ Maturity + Sector + (1 \| ID) | 9 | 6.49x10^-13^ | -20.71 | 3.39x10^-13^ | 19.82 |
| ~ Maturity + BC + Sector + (1 \| ID) | 11 | 1.06x10^-13^ | -17.08 | 5.53x10^-14^ | 20.24 |
| ~ Sex + Maturity + Sector + (1 \| ID) | 11 | 8.84x10^-14^ | -16.72 | 4.61x10^-14^ | 20.06 |
| ~ Sex + Maturity + BC + Sector + (1 \| ID) | 13 | 1.42x10^-14^ | -13.07 | 7.42x10^-15^ | 20.50 |
| ~ Sector + (1 \| ID) | 7 | 4.88x10^-27^ | 44.33 | 2.55x10^-27^ | -14.88 |
| ~ Sector + Sex + (1 \| ID) | 9 | 1.37x10^-27^ | 46.87 | 7.16x10^-28^ | -13.97 |
| ~ BC + Sector + (1 \| ID) | 9 | 5.95x10^-28^ | 48.54 | 3.11x10^-28^ | -14.80 |
| ~ BC + Sex + Sector + (1 \| ID) | 11 | 1.66x10^-28^ | 51.09 | 8.68x10^-29^ | -13.85 |

**Table S9** Performance values for zero-inflated generalised linear mixed models (ZIGLMMs) analysing cranial versus caudal sector groupings in common dolphins (Delphinus delphis) assessed postmortem. These models incorporate sex (male or female), sector (sector grouping; cranial or caudal), maturity (sexual maturity; immature or mature), age (years), TBL (total body length, cm), BC (body condition; good, moderate, or poor), and ID (individual identity) to estimate the rake density (rm/dm^2^) of each individual. Note: k = number of parameters, LogLik = minimized negative log-likelihood, AICc = corrected Akaike Information Criterion for small sample sizes, wAICc = Akaike weight adjusted to sum up to 1, ER = evidence ratio, calculated in comparison to the best-performing model.

| Model | k | ER | AICc | wAICc | LogLik |
| --- | --- | --- | --- | --- | --- |
| Rake density (cranial vs caudal) |  |  |  |  |  |
| ~ Sex + Age + TBL * Sector + (1 \| ID) | 15 | - | -40.95 | 0.84 | 36.77 |
| ~ Age + TBL + Sector + (1 \| ID) | 11 | 0.04 | -34.29 | 0.03 | 28.84 |
| ~ Sex + Age + TBL + Sector + (1 \| ID) | 13 | 0.04 | -34.27 | 0.03 | 31.10 |
| ~ Sector + Age + Maturity + TBL + (1 \| ID) | 13 | 0.03 | -33.85 | 0.02 | 30.89 |
| ~ TBL + Sector + (1 \| ID) | 9 | 0.03 | -33.80 | 0.02 | 26.37 |
| ~ Sex + TBL + Sector + (1 \| ID) | 11 | 0.03 | -33.60 | 0.02 | 28.49 |
| ~ Sex + Age + Maturity + TBL + Sector + (1 \| ID) | 15 | 0.02 | -32.96 | 0.02 | 32.77 |
| ~ Sex + TBL + Sector + (1 \| ID) | 11 | 0.00 | -30.16 | 0.00 | 26.77 |
| ~ Sex + Age + Maturity + TBL + Sector + (1 \| ID) | 13 | 0.00 | -29.33 | 0.00 | 28.63 |
| ~ Maturity + TBL + Sector + (1 \| ID) | 17 | 0.00 | -27.08 | 0.00 | 32.20 |
| ~ Maturity + Sex + TBL + Sector + (1 \| ID) | 15 | 0.00 | -26.82 | 0.00 | 29.70 |
| ~ Sex + Age + BC + TBL + Sector + (1 \| ID) | 15 | 0.00 | -26.64 | 0.00 | 29.61 |
| ~ Sex + Age + BC + TBL + Sector + (1 \| ID) | 13 | 0.00 | -26.58 | 0.00 | 27.26 |
| ~ Sex + TBL + BC + Sector + (1 \| ID) | 17 | 0.00 | -26.04 | 0.00 | 31.68 |
| ~ Age + TBL + BC + Sector + (1 \| ID) | 19 | 0.00 | -25.47 | 0.00 | 33.82 |
| ~ BC + TBL + Sector + (1 \| ID) | 11 | 0.00 | -22.86 | 0.00 | 23.12 |
| ~ BC + Age + Maturity + TBL + Sector + (1 \| ID) | 15 | 0.00 | -22.77 | 0.00 | 27.68 |
| ~ Age + Maturity + Sex + BC + TBL + Sector + (1 \| ID) | 17 | 0.00 | -22.28 | 0.00 | 29.80 |
| ~ Maturity + Age + Sector + (1 \| ID) | 13 | 0.00 | -19.62 | 0.00 | 23.78 |
| ~ Sex + Age + Maturity + Sector + (1 \| ID) | 14 | 0.00 | -19.13 | 0.00 | 24.69 |
| ~ Sex + TBL + Maturity + BC + Sector + (1 \| ID) | 9 | 0.00 | -16.83 | 0.00 | 17.88 |
| ~ Age + Sex + Maturity + Sector + (1 \| ID) | 11 | 0.00 | -14.46 | 0.00 | 18.92 |
| ~ Age + Maturity + BC + Sector + (1 \| ID) | 17 | 0.00 | -12.40 | 0.00 | 24.86 |
| ~ Age + BC + Sector + (1 \| ID) | 13 | 0.00 | -9.78 | 0.00 | 18.86 |
| ~ Sex + Age + BC + Sector + (1 \| ID) | 15 | 0.00 | -7.77 | 0.00 | 20.18 |
| ~ Sector + Maturity + (1 \| ID) | 9 | 0.00 | 12.02 | 0.00 | 3.46 |
| ~ Sex + Maturity + Sector + (1 \| ID) | 11 | 0.00 | 15.35 | 0.00 | 4.02 |
| ~ BC + Maturity + Sector + (1 \| ID) | 13 | 0.00 | 18.32 | 0.00 | 4.81 |
| ~ Sex + Maturity + BC + Sector + (1 \| ID) | 15 | 0.00 | 21.60 | 0.00 | 5.49 |
| ~ Sector + (1 \| ID) | 7 | 0.00 | 72.36 | 0.00 | -28.89 |
| ~ Sector + Sex + (1 \| ID) | 9 | 0.00 | 72.90 | 0.00 | -26.98 |
| ~ BC + Sector + (1 \| ID) | 11 | 0.00 | 77.78 | 0.00 | -27.19 |
| ~ Sex + BC + Sector + (1 \| ID) | 13 | 0.00 | 78.24 | 0.00 | -25.15 |

**FIGURES**

**Figure S1** Methods for calculating body sector areas of common dolphins (*Delphinus delphis*): Blue triangles – triangle area formula; Pink rectangles – rectangle area formula; Green ovals – Adobe Photoshop pixel tracing.

**Figure S2** Supplementary scanned rake mark data tables showing lesion characteristics associated with rake mark diagrams in figure 2 recorded during postmortem assessments, including rake length, rake width, and inter-rake distance in common dolphins (Delphinus delphis) for individuals with (a) low rake density (0.02 rm/dm^2^), (b) medium rake density (0.46 rm/dm^2^), and (c) high rake density (1.06 rm/dm^2^).

**Figure S3** The relationship between rake density (rm/dm^2^) and age (years) in common dolphins (*Delphinus delphis*) assessed during postmortem examinations, between 2006 and 2024 in New Zealand. Each blue dot represents an individual dolphin’s age and corresponding rake density. The pink linear regression line indicates the general trend, showing changes in rake density with age.

**Figure S4** The relationship between rake density (rm/dm^2^) and total body length (TBL, in cm) in common dolphins (*Delphinus delphis*) assessed during postmortem examinations between 2006 and 2024 in New Zealand. Each blue dot represents an individual dolphin’s TBL and corresponding rake density. The pink linear regression line indicates the general trend, showing how changes in rake density with TBL.

**REFERENCES**

Stockin, K. A., Duignan, P. J., Roe, W. D., Meynier, L., Alley, M., & Fettermann, T. (2009). Causes of mortality in stranded common dolphin (*Delphinus* sp.) from New Zealand waters between 1998 and 2008. *Pacific Conservation Biology, 15*(3), 217-227. https://doi.org/10.1071/PC090217

IJsseldijk, L. L., Brownlow, A. C., & Mazzariol, S. (2019). European best practice on cetacean post-mortem investigation and tissue sampling. <https://doi.org/10.31219/osf.io/zh4ra>
